# Editorial: New Advances in RNA Targeting

**DOI:** 10.3389/fphar.2020.00468

**Published:** 2020-04-17

**Authors:** Olga Ilinskaya, Derek J. Hausenloy, Hector A. Cabrera-Fuentes, Marina Zenkova

**Affiliations:** ^1^ Institute of Fundamental Medicine and Biology, Kazan (Volga Region) Federal University, Kazan, Russia; ^2^ Cardiovascular & Metabolic Disorders Program, Duke-National University of Singapore Medical School, Singapore, Singapore; ^3^ National Heart Centre, National Heart Research Institute Singapore, Singapore, Singapore; ^4^ Yong Loo Lin School of Medicine, National University Singapore, Singapore, Singapore; ^5^ The Hatter Cardiovascular Institute, University College London, London, United Kingdom; ^6^ Cardiovascular Research Center, College of Medical and Health Sciences, Asia University, Taichung, Taiwan; ^7^ Institute of Biochemistry, Justus-Liebig-University Giessen, Giessen, Germany; ^8^ Laboratory of Biochemistry of Nucleic Acids, Institute of Chemical Biology and Fundamental Medicine of Russian Academy of Science, Novosibirsk, Russia

**Keywords:** oligonucleotides, noncoding RNAs, RNases, bioconjugates, delivery

Recent discoveries have implicated specific genes in the pathogenesis of a variety of cancers, neuronal and infectious diseases, and generating a new trend in drug development. As such, scientists have been searching for new ways to turn off the target gene(s) causing the disease without affecting the DNA sequence itself. Figuratively speaking, this therapeutic approach needs “bow and arrows” to hit the RNA target. The main concept of RNA-targeting therapy is the use of molecular “arrows” selectively hitting the structure and functional activity of specific RNAs to alter the expression of disease-relevant genes. A large number of investigational RNA-targeting drugs are currently under clinical development, although clinical translation has yet to be realized. This means that manipulating the functions and structures of such complex and dynamic targets as RNA requires a detailed analysis of existing approaches and the development of novel ones.

In this Research Topic *Frontiers in Pharmacology*, are several studies investigating RNA-targeted therapeutics involving chemically modified oligonucleotides [antisense oligonucleotides, aptamers, small interfering RNAs (siRNAs), microRNAs (miRNAs), and synthetic messenger RNAs (mRNAs)], a wide range of small molecules inhibiting interactions between protein and RNA, and RNases of different origin. New alternatives to antibiotics are now in great demand for the effective treatment of microbial infections. Novopashina et al. have developed novel oligonucleotides to inhibit RNase P, an essential bacterial enzyme, to inhibit bacterial growth, thereby providing a novel therapeutic for combating microbial infections. Patutina et al. have designed a novel platform for knockdown of miRNA targets, which is based on synthetic, sequence-specific ribonucleases. MiRNases (peptides capable of RNA cleavage) can be conjugated to miRNA-targeted oligodeoxyribonucleotides, rendering them resistant to nuclease-resistant within the conjugate design, without resorting to chemically-modified nucleotides. This miRNase-design platform can be easily adapted to therapeutically target pathogenic microRNAs that are overexpressed in many diseases.

There are several examples in which the analysis of transcriptomic datasets have provided insights into underlying disease mechanisms, disease epidemiology, and have the potential to discover new RNA gene targets for treating a wide variety of medical conditions. Tezcan et al. provide a comprehensive overview on the role of microRNA post-transcriptional regulation of the NLRP3 inflammasome in different immune related conditions. This has been shown to be effective in animal models but whether this can be translated into the clinical setting remains to be determined. Khaiboullina et al. have examined transcriptomic datasets of human umbilical vein endothelial cells (HUVECs) following Zika infection and identified several potential cytokine mediators of endothelial permeability. A bioinformatic study by Kuznetsov et al. investigated the changes in the transcriptome which occurred in the mouse lumbar spinal cord after a 30-day space flight and 7-day re-adaptation period on Earth, and reported new insights into the mechanisms underlying the hypogravity motor syndrome. In an interesting article by Tarlinton et al., analysis of peripheral monocyte gene expression found a correlation between regional changes in expression of human endogenous retrovirus W (HERV-W) and the prevalence of multiple sclerosis between different ethnic populations in Britain and the republic of Tatarstan. In a similar approach, Davidyuk et al. have investigated the relationship between the presence of *Puumala orthohantavirus* strains in small animals captured in the Republic of Tatarstan and Finland, and correlated the findings with the clinical features of hemorrhagic fever with renal syndrome.

Although synthetic oligonucleotides can be designed to bind to target RNA and modify the latter's function, the broader potential of these compounds as therapeutics has remained untapped because their delivery to cells has been limited. In this regard, Skvortsova et al. have demonstrated that antisense oligonucleotide derivatives can be used to target gene expression and inhibit the growth of intracellular mycobacteria. In this study they showed that the new RNA analogue, phosphoryl guanidine oligo-2'-o-methylribonucleotide, could be efficiently taken up by intracellular microorganisms with strong antisense activity, thereby providing a new treatment strategy for tuberculosis, and potentially preventing the emergence of drug-resistant strains of mycobacteria.

Most of the human genome encodes RNA that do not code for protein. Noncoding RNAs may modulate gene expression and onset and progression of disease, positioning them as new therapeutic targets for drug discovery. Miroshnichenko and Patutina, provide an overview of review one of the different approaches for regulating the function of short noncoding RNAs, particularly miRNAs. The latter are viable targets for anticancer therapeutic, given that miRNAs play a key role in modulating a large number of signaling pathways involved with cell proliferation, apoptosis, migration, and invasion. Anticancer therapy using antisense oligonucleotide constructs have been shown to control miRNA activity, and these include a variety of strategies such as small RNA zippers, miRNases, miRNA sponges, miRNA masks, anti-miRNA oligonucleotides, and synthetic miRNA mimics. Furthermore, small RNA zipper technology may be utilized to ablate function of endogenous siRNAs and Piwi-interacting RNAs (piRNAs).

In the last few years, CRISPR–Cas systems have been introduced as a powerful mode of RNA-editing strategy, that provides an important alternative to DNA editing which can cause so called “off-target effects”—unwanted mutations in other parts of the genome. Filippova et al. have shown that small nucleolar RNAs (snoRNA) in human cells can be gene edited using CRISPR/Cas9 cleavage.

Over many years, RNases have been investigated as potential antitumor agents given their selectivity and toxicity against certain transformed cells. However, the mechanisms underlying their selective cytotoxic effects remain unclear, and may include controlling RNA hydrolysis products, and selective suppression of specific genes. Elucidating the underlying mechanisms requires understanding of the transcriptome of RNAase treated cells. In this regard, exogenous RNases can modify the redox potential of key proteins (e.g., NF-kB, p53) by suppressing reactive oxygen species (ROS) production in tumor cells, thereby increasing the susceptibility of cancer cells to apoptotic cell death and attenuating uncontrolled division of cancer cells. In most situations, the cytotoxic efficacy of RNases is dependent on their ability to be taken up by the cancer cells. Mitkevich et al. provide an overview of the potential role of exogenous RNases in mediating the adaptive response of tumor cells which allow the latter to remain active despite changes to the micro-environment including acidic and hypoxic factors. Mironova and Vlassov describe a large number of tumor-associated intracellular RNAs and extracellular RNAs, which can be targeted by exogenous RNAases, as therapeutic strategies for treating a variety of different tumors.

Prats-Ejarque et al. have analyzed the RNase A superfamily using kinetic assays and molecular dynamics simulations to identify the structural motifs for nucleotide recognition in RNases which make up the host defense, thereby providing a strategy for structure-based drug discovery.

Several articles have addressed the problem of delivering RNA-targeting therapeutics into diseased cells. In order to find an effective “bow” to direct the therapeutic agent to the desired cellular target, novel approaches are needed. Conjugating therapeutics with antibodies that have the ability to recognize cell-specific surface receptors can be employed to target drugs to particular cancer cells, but this technology has a number of limitations. Nanoparticle-delivery of therapeutics has emerged as an alternative approach to deliver RNA-targeting drugs. In this regard, Chernikov et al. used bioconjugation, which is the covalent binding of siRNAs with biogenic molecules (such as lipophilic proteins, aptamers, antibodies, ligands, peptides, or polymers). Bioconjugates make very good nanoparticles as they do not require a positive charge to form complexes, are less recognized by components of the immune system, and are less cytotoxic because of their small size. Markov et al. have reviewed the role of exosomes as an alternative to synthetic nanoparticles. Extracellular vesicles may be used as natural vectors for delivery of RNA and other therapeutics targeted to tumor cells, T-lymphocytes, and dendritic cells. Therefore, extracellular vesicles have the therapeutic potential to be used as novel cell-free anti-tumor vaccines providing an alternative to dendritic cell-based vaccines. Chinak et al. have shown that cell-penetrating peptides may be used to transport cargo into cells. They were able to show that non-covalently associated nucleic acids could be delivered into cancer cells *in vitro* using recombinant protein lactaptin.

Khojaewa et al. have explored the potential of natural and synthetic zeolites to deliver the RNase, binase, as a potential antitumor drug. They used a simple approach based on immobilizing the antitumor RNase on natural minerals of the zeolite group. Bacterial RNase were shown to complex with clinoptilolite and this increased cytotoxicity, a therapeutic approach which can applied using zeolitezeolite-based complexes with RNA-targeting therapeutics for treatment of colorectal cancer, and when combined in a cream it can be used to treat malignant skin neoplasms.

Well-tolerated humans vaccines based on viral mRNAs with optimized sequences have the therapeutic potential to treat infectious diseases, effective protein translation, and stimulation of immune response can persist for several days. Furthermore, the safety of vaccines can be provided by cellular RNases that have the ability to target viral mRNA. It is also necessary to pay attention to the identified antiviral potential of bacterial RNases: exogenous RNase from *Bacillus pumilus* has been shown to inhibit the replication of Middle East respiratory syndrome-related coronavirus (MERS-CoV) and human coronavirus 229E (HCoV-229E) (Müller et al., 2017). This raises the possibility of using mRNA-based vaccines as well as bacterial RNases to combat against the current COVID-19 pandemic.

In the near future, tasks to make the RNA-targeting molecules more potent and less immunogenic as well as to increase their delivery and prolonged action should be pursued.

## Author Contributions

DH and OI wrote this article. HC-F and MZ have made a direct and intellectual contribution to the work. All authors have approved the article for publication.

## Funding

OI was supported by Russian Government Program of Competitive Growth of Kazan University and RFBR project No. 17-00-00060, MZ was supported by RFBR project No. 17-00-00062 (K). DH was supported by the British Heart Foundation (CS/14/3/31002), Singapore MHNMR Council (NMRC/CSA-SI/0011/2017) and Collaborative Centre Grant scheme (NMRC/CGAug16C006), and the Singapore MEAR Fund Tier 2 (MOE2016-T2-2-021). This article is based upon work from COST Action EU-CARDIOPROTECTION CA16225 supported by COST.

## Conflict of Interest

The authors declare that the research was conducted in the absence of any commercial or financial relationships that could be construed as a potential conflict of interest.
